# Primary Pulmonary Choriocarcinoma With Lymph Node Metastasis: A Case Report and Brief Review

**DOI:** 10.7759/cureus.73010

**Published:** 2024-11-04

**Authors:** Chinedu Okoli, Brianna Philbrick, Syed Quadri

**Affiliations:** 1 General Surgery, MaineHealth, Portland, USA; 2 General Surgery, Maine Medical Center, Portland, USA; 3 Thoracic Surgery, Maine Medical Center, Portland, USA

**Keywords:** chemotherapy, lymph node metastasis, primary pulmonary choriocarcinoma, wedge resection, β-hcg

## Abstract

Choriocarcinoma is a malignant germ cell tumor containing syncytiotrophoblasts and secreting human chorionic gonadotropin (β-hCG), often associated with a poor prognosis. Reports of primary choriocarcinoma of the lung with lymph node metastasis are extremely rare in the literature. Here, we report a surgically treated case of primary pulmonary choriocarcinoma in a 32-year-old woman. Surgery was followed by three cycles of chemotherapy with etoposide, methotrexate, actinomycin D, leucovorin calcium, cyclophosphamide, and vincristine. Her post-treatment PET scan showed no evidence of disease, and her β-hCG remains at nadir level. She is currently on monthly hCG surveillance and is in good condition.

## Introduction

Gestational trophoblastic disease (GTD) is a group of tumors defined by abnormal trophoblastic proliferation, encompassing both benign and malignant entities. Histologically, GTD is divided into molar (containing villi) and non-molar neoplasms (lacking villi). The molar forms include partial hydatidiform mole (PHM) and complete hydatidiform mole (CHM), while the non-molar forms consist of invasive mole, choriocarcinoma, epithelioid trophoblastic tumor (ETT), and placental-site trophoblastic tumor (PSTT). Among the various types of non-molar GTDs, choriocarcinoma is the most malignant form and is highly prone to disseminated metastasis [1]. It comprises three morphologically distinct tissues: human chorionic gonadotropin (hCG)-producing multinucleated syncytiotrophoblasts, intermediate trophoblasts, and cytotrophoblasts [2]. Primary extragenital choriocarcinoma is uncommon and has been reported in various organs, including the liver [3], colon [4], urinary bladder [5], stomach [6], and lungs [7]. Since the lung is a common site of choriocarcinoma metastasis, a thorough search for an occult primary tumor is necessary. The clinical symptoms of primary pulmonary choriocarcinoma (PPC) are non-specific and can easily be mistaken for more common conditions, such as ectopic pregnancy, as seen in our case.

In general, gestational choriocarcinoma has a better prognosis than non-gestational choriocarcinoma, even in cases of disseminated disease [8]. In contrast, the natural course of PPC is associated with a poor prognosis. The reasons for the differences in behavior between non-gestational and gestational choriocarcinoma remain unknown. A review of the literature indicates that most cases of PPC are treated with a combination of surgery and chemotherapy. The preferred chemotherapy regimen in most countries is EMA-CO (etoposide, methotrexate, actinomycin D, cyclophosphamide, and vincristine) [9]. While the role of surgery in managing PPC is well established, there is limited data on the optimal surgical approach, specifically whether lobectomy or wedge resection is more effective. Furthermore, it remains unclear whether systematic or selective lymph node (LN) dissection offers any benefits.

Here, we present a case of a solitary lung tumor with left hilar LN metastasis, identified through cross-sectional imaging. We will also discuss the diagnosis and treatment of this case, along with a brief review of the literature.

## Case presentation

A 32-year-old female presented with persistently elevated hCG levels and a suspicion of GTD. Her obstetrics and gynecology history included a term delivery seven months prior to this clinical presentation. She reported experiencing vaginal bleeding following a period of amenorrhea while breastfeeding. She had a positive home pregnancy test and subsequently developed vaginal bleeding the following month. Initially, this was suspected to be a miscarriage, but her hCG levels continued to rise. An ultrasound was performed at the time of presentation, which did not demonstrate an intrauterine pregnancy. She was treated with methotrexate, 50 mg/m², for a presumed ectopic pregnancy. Serial hCG measurements then showed a slow decline, prompting a repeat ultrasound a month later. This scan still showed no evidence of an intrauterine pregnancy.

**Figure 1:**
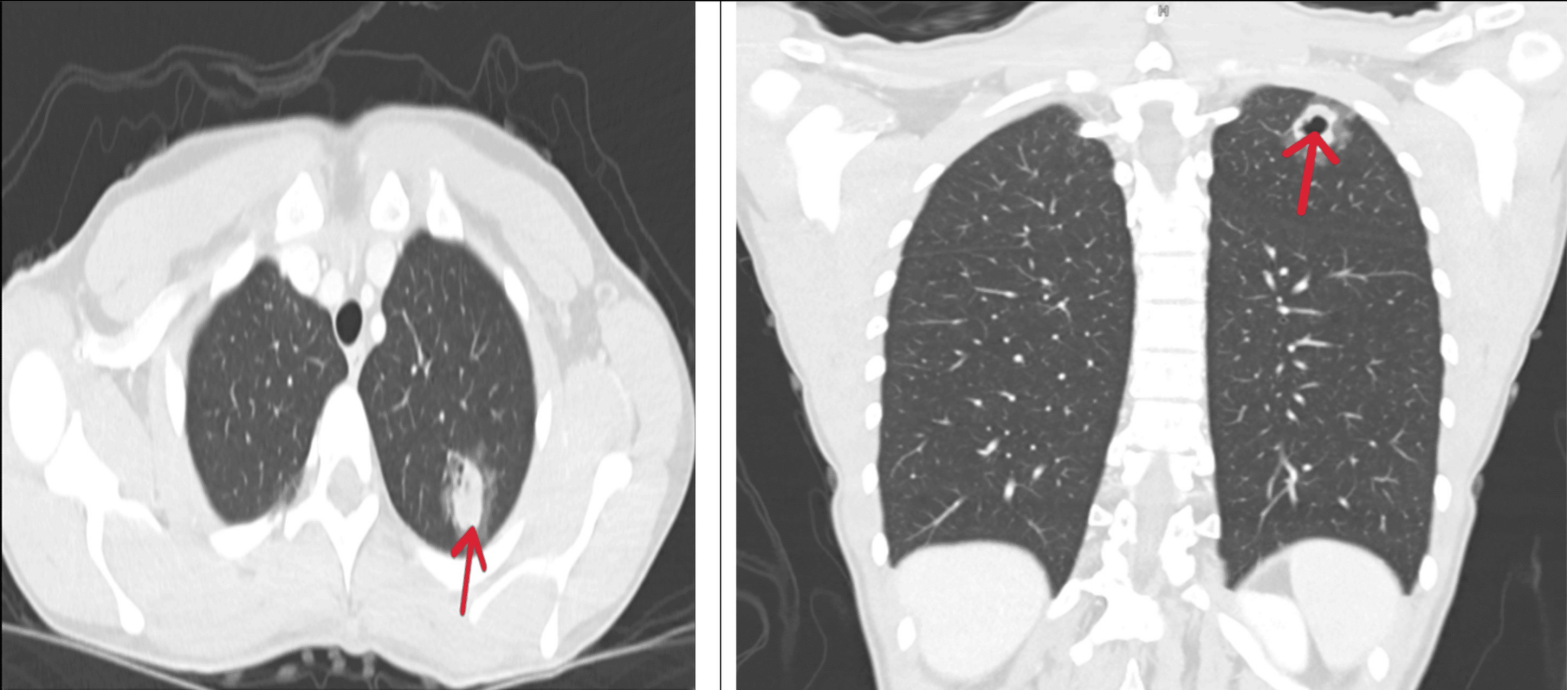
CT scan showing the left apical mass: axial and coronal views

She was administered a second dose of methotrexate, 50 mg/m², for ectopic pregnancy versus pregnancy of unknown location. Due to persistently elevated hCG levels, she underwent diagnostic laparoscopy to evaluate for ectopic pregnancy. She also underwent chromopertubation and hysteroscopy with dilation and curettage. There was no gross evidence of an ectopic pregnancy on laparoscopy, and her fallopian tubes were patent. Pathology from the collected specimen revealed proliferative phase endometrium without chorionic villi. At this point, a tentative diagnosis of GTD was made. A second opinion from another provider led to additional imaging studies, including computed tomography (CT) of the chest, abdomen, and pelvis. The CT of the chest revealed a cavitary lung mass in the left upper lobe (see Figure 1).

Notably, she did not have any pulmonary symptoms or complaints; she denied chest pain, fevers, chills, sweats, or hemoptysis. She was subsequently referred to thoracic surgery for consultation regarding the left upper lobe lung nodule. A PET CT scan showed moderately intense peripheral uptake in the cavitary left upper lobe mass, likely indicating malignancy, along with an intensely FDG-avid left hilar LN, raising suspicion for nodal metastatic disease. At this point, she was determined to have stage III disease with a WHO score of 6. She then underwent a flexible bronchoscopy, robotic left thoracoscopy, and left upper lobe wedge resection. After surgery, her hCG levels dropped dramatically from 405 mIU/mL to 178.9 mIU/mL on postoperative day one (values less than 5.3 mIU/mL are consistent with nonpregnant females) and subsequently to <1 mIU/mL four weeks after surgery, before chemotherapy was initiated (see Appendices for the laboratory reference range). See Table 1 for the molecular typing of the tumor.

**Table 1:** Molecular typing of the tumor SALL: Spalt-like gene family, AFP: alpha-fetoprotein.

Molecular marker	Status
Cytokeratin AE1/3	Positive
Glypican	Focal positive
SALL4	Positive
Inhibin	Focal positive
CD30	Negative
AFP	Negative

**Figure 2:**
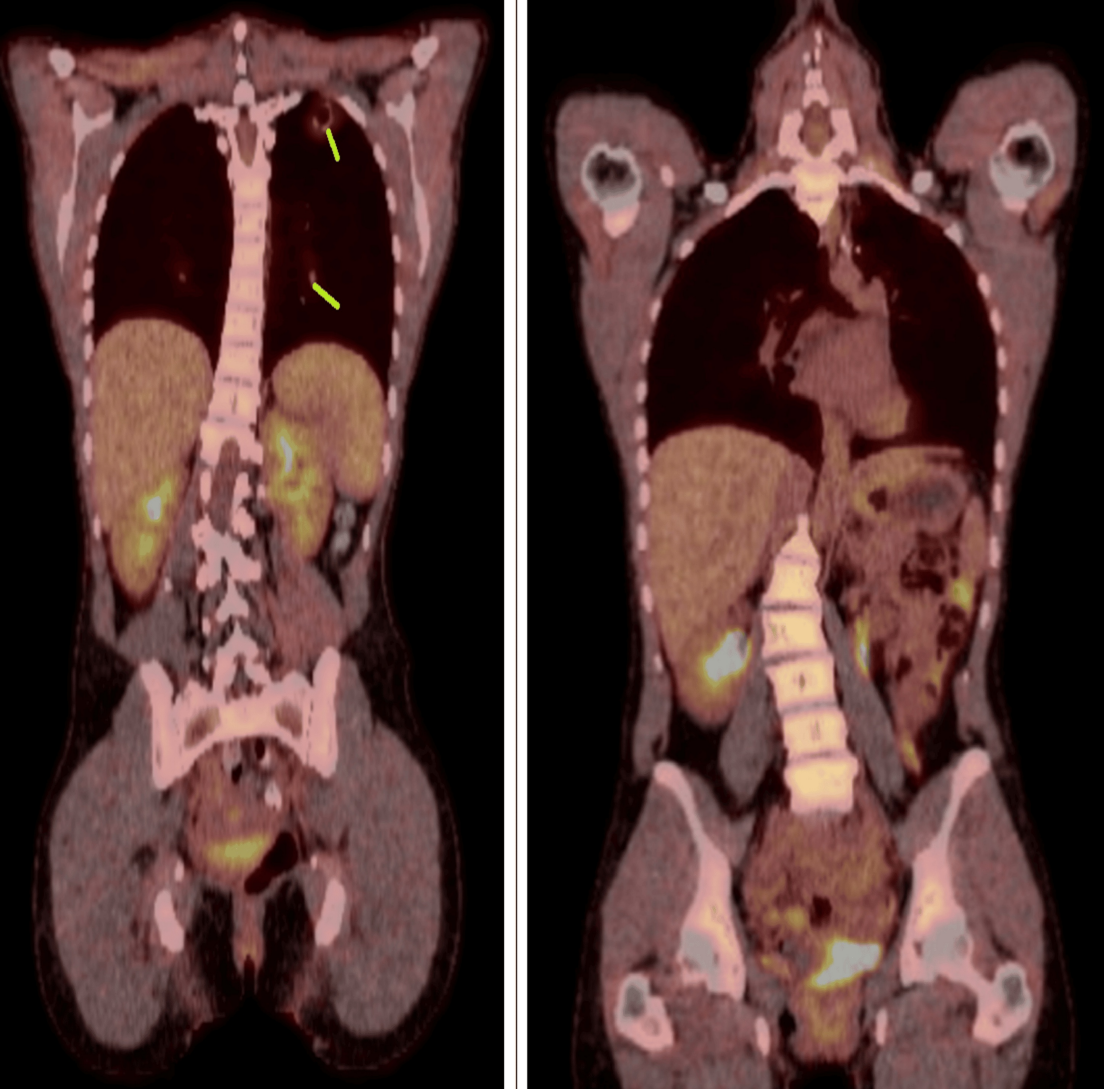
PET scan, pre-treatment (left) vs post-treatment (surgery and chemotherapy) (right)

Because of her stage III disease, adjuvant primary multiagent chemotherapy, including EMA-CO (etoposide, methotrexate, actinomycin D, leucovorin calcium, cyclophosphamide, and vincristine), was recommended by the multidisciplinary tumor board. Before beginning chemotherapy, she had a ParaGard IUD (copper intrauterine device) placed and started on Depo Provera. She also underwent egg harvesting and preservation. She completed three cycles of chemotherapy, which she tolerated well, given the PET/CT findings of possible LN metastasis. Repeat PET/CT findings after the third chemotherapy cycle showed no evidence of disease, as shown in Figure 2.

Her hCG level was measured before each chemotherapy cycle, and it remained <1 mIU/mL. She is currently undergoing monthly hCG surveillance for 12 months, with the option for a repeat chest CT at the end of the surveillance period.

## Discussion

Choriocarcinoma is a rare malignant germ cell tumor that develops from an abnormal trophoblastic population undergoing hyperplasia and anaplasia [10]. Gestational choriocarcinoma arises from pluripotent germ cells [8]. The exact pathogenesis of pulmonary choriocarcinoma has not been fully explained. Four hypotheses have been proposed for the development of PPC. The first hypothesis is that PPC may develop from metastasis of primary gonadal choriocarcinoma that regressed spontaneously [11]. The so-called “burnout” phenomenon is a unique and specific feature of choriocarcinoma. This is thought to be due to the rejection of tumors of trophoblast origin at any point in the progression of the disease due to the genetic disparity between the maternal host and the tumor tissue of fetal origin, as the fetus possesses both maternal and fetal antigens-just as there is a natural tendency for the rejection of the trophoblast of a normal pregnancy culminating either in parturition or spontaneous abortion [11]. The second hypothesis is that PPC may have originated from a trophoblastic embolus related to a gestational event after a long latency period associated with spontaneous regression of the primary gonadal choriocarcinoma [12]. Schmorl first reported the embolization of large numbers of detached syncytial cells to the maternal lung in women dying from eclampsia [12]. Since then, it has been shown to represent a normal phenomenon during all human pregnancies [13]. The third hypothesis about the origin of PPC postulates that it may originate from retained primordial germ cells that migrated abnormally during embryogenesis [14]. Finally, PPC may arise from the de-differentiation or trans-differentiation of lung cancer that develops initially as a non-trophoblastic neoplasm [15]. It is noteworthy that immunohistochemistry analysis using beta-hCG, Ki-67, cytokeratin, placental alkaline phosphatase (PLAP), and CD30 can help physicians make differential diagnoses [16].

Our case presentation showed pulmonary choriocarcinoma with suspected hilar LN metastasis (based on PET findings but not biopsy-proven), and the diagnosis was delayed because the patient had limited pulmonary symptoms. Often, the diagnosis is made after cross-sectional whole-body imaging and confirmed after final surgical management. Her hCG fell dramatically after the wedge resection of the lesion, and the patient’s clinical course after surgery was uneventful. During her diagnostic laparoscopy, the ovaries, uterus, and uterine tubes were found to be free of lesions. The histological examination of our patient’s tumor showed only the characteristic two populations of tumor cells. Thus, the present case met clinical, cytological, histological, and immunohistochemical criteria for diagnosis of PPC of the lung.

We conducted a literature review and identified 68 reported cases of PPC. Most patients presented with pulmonary symptoms such as cough, hemoptysis, chest pain, or dyspnea, although 17 other cases did not have these presenting symptoms. There are no established guidelines for treating PPC, so it is generally managed according to primary gonadal choriocarcinoma treatment protocols. Due to the undifferentiated nature of the malignancy, PPC responds poorly to radiotherapy [7]. Surgery followed by adjuvant chemotherapy appears to be the best treatment modality to date. Based on our review, LN metastasis is uncommon, with only two cases reported, consistent with the typical hematogenous spread of choriocarcinoma [17].

While the five-year mortality rate for PPC is extremely low (under 5%), our patient has a better prognosis, as women with a history of gestational events appear to have improved outcomes compared to those without such a history [18]. However, in the absence of characteristic immunohistochemical features of PPC, it remains controversial whether women with pregnancy events have distinct pathophysiological features or whether occult gestational choriocarcinoma with pulmonary metastasis is misdiagnosed as PPC, leading to a good survival outcome on statistical analysis.

Although the literature largely agrees that surgery combined with chemotherapy is indicated for PPC, the role of wedge resection versus lobectomy remains unclear. This is particularly true for our patient, who may have ipsilateral LN metastasis, which, based on her anatomy, would only be accessible for sampling via completion lobectomy. Studies comparing wedge resection to lobectomy for other forms of lung cancer have demonstrated the benefits of wedge resection, including lower complication rates and shorter hospital stays [19]. We propose that wedge resection combined with chemotherapy may be an optimal treatment approach in selected cases, as the patient’s hCG level dropped to nadir following the wedge resection. However, long-term follow-up will be necessary to confirm this as a recommended treatment strategy.

## Conclusions

In summary, PPC is a rare disease with varied clinical characteristics. A review of the literature suggests that surgical resection followed by adjuvant chemotherapy is the most effective treatment for this condition. However, the roles of wedge resection versus lobectomy remain unclear, and the benefits of LN dissection are still unknown.

## Appendices

Reference ranges for human chorionic gonadotrophin (hCG) for the laboratory are presented in Table 2.

**Table 2:** Reference ranges for human chorionic gonadotrophin (hCG) for the laboratory Values less than 5.3 mIU/mL are consistent with nonpregnant females. Mildly elevated values (5–25 MIU/mL) have been observed in postmenopausal women.

Weeks Gestation	Range (mIU/mL)
3	5.8–71.2
4	9.5–750
5	217–7,138
6	158–31,795
7	3,697–163,563
8	32,065–149,571
9	63,803–151,410
10	46,509–186,977
12	27,832–210,612
14	13,950–62,530
15	12,039–70,971
16	9,040–56,451
17	8,175–55,868
18	8,099–58,176
